# Enhanced apoptosis as a possible mechanism to self-limit SARS-CoV-2 replication in porcine primary respiratory epithelial cells in contrast to human cells

**DOI:** 10.1038/s41420-021-00781-w

**Published:** 2021-12-10

**Authors:** Rahul K. Nelli, Kruttika-S Phadke, Gino Castillo, Lu Yen, Amy Saunders, Rolf Rauh, William Nelson, Bryan H. Bellaire, Luis G. Giménez-Lirola

**Affiliations:** 1 Department of Veterinary Diagnostic and Production Animal Medicine, College of Veterinary Medicine, Iowa State University, Ames, IA 50010 USA; 2 Department of Veterinary Microbiology and Preventive Medicine, College of Veterinary Medicine, Iowa State University, Ames, IA 50010 USA; 3 Tetracore, Inc., Rockville, MD 20850 USA

**Keywords:** Microbiology, Immune cell death

## Abstract

The ability of SARS-CoV to infect different species, including humans, dogs, cats, minks, ferrets, hamsters, tigers, and deer, pose a continuous threat to human and animal health. Pigs, though closely related to humans, seem to be less susceptible to SARS-CoV-2. Former in vivo studies failed to demonstrate clinical signs and transmission between pigs, while later attempts using a higher infectious dose reported viral shedding and seroconversion. This study investigated species-specific cell susceptibility, virus dose-dependent infectivity, and infection kinetics, using primary human (HRECs) and porcine (PRECs) respiratory epithelial cells. Despite higher ACE2 expression in HRECs compared to PRECs, SARS-CoV-2 infected, and replicated in both PRECs and HRECs in a dose-dependent manner. Cytopathic effect was particularly more evident in PRECs than HRECs, showing the hallmark morphological signs of apoptosis. Further analysis confirmed an early and enhanced apoptotic mechanism driven through caspase 3/7 activation, limiting SARS-CoV-2 propagation in PRECs compared to HRECs. Our findings shed light on a possible mechanism of resistance of pigs to SARS-CoV-2 infection, and it may hold therapeutic value for the treatment of COVID-19.

## Introduction

The ability to infect a wide variety of mammalian and avian species [1], coronavirus (order *Nidovirales*, suborder *Cornidovirineae*, family *Coronaviridae*, subfamily *Orthocoronavirinae*) infections pose a recurring and continuous threat to human and animal health, particularly the new viral strains emerging from unknown wild animal reservoirs [2–4]. Historically, coronavirus infections in humans (e.g., those caused by HCoV-229E and HCoV-OC43 CoV strains) are mild and associated with only common cold symptoms [5–7]. However, the emergence of the betacoronaviruses, severe acute respiratory syndrome virus (SARS-CoV) (case-fatality rate: 9.6%) [8, 9], Middle East respiratory syndrome virus (MERS-CoV) (case-fatality rate: 34.4%) [10, 11], and most recently, SARS-CoV-2 (case fatality rate: 2%) [12, 13], the specific origin of which remains elusive and under continuous debate, have demonstrated the potential of coronaviruses to cause fatal disease in humans.

The COVID-19 pandemic highlights the importance of improving our understanding of how SARS-CoV-2 causes disease and spreads beyond humans. It also stimulated intensive research towards developing animal models, with a particular focus on the angiotensin-converting enzyme 2 (ACE2), which is the host cell receptor for SARS-CoV-2. Remarkably, different studies have demonstrated that several animal species, including dogs [14], cats [15], minks [16], ferrets [17], hamsters [18], tigers [19], and deer [20], are susceptible to infection by SARS-CoV-2 through zoo-anthroponotic (or reverse-zoonotic) transmission [21].

Interestingly, there is no known incidence of SARS-CoV-2-associated disease in pigs, and different experimental studies in pigs have failed to show clinical signs and transmission between animals [14, 22–25]. Former experimental studies in pigs showed no detection of viral RNA in nasal swabs or tissues nor seroconversion in response to SARS-CoV-2 inoculation [14, 22, 23]. However, a later study that used a higher inoculating dose than the previous studies reported mild pig susceptibility to SARS-CoV-2 as demonstrated by viral RNA and antibody detection in oral fluids and nasal wash in some inoculating pigs [25]. Moreover, Vergara-Alert and others observed SARS-CoV-2 neutralizing antibody response in pigs inoculated parenterally [24], suggesting that pigs could be used as a model for viral immunogenicity studies. Understanding that host susceptibility is affected by many factors, and considering the complexity of reproducing infection under in vivo experimental conditions, current evidence suggests that pigs are comparatively less susceptible or more resistant to SARS-CoV-2 than humans and some other animal species (e.g., mink, deer) [16, 20]. However, the factors responsible for the lack of virus susceptibility or virus replication in pigs remain uninvestigated. This in vitro study evaluated differences in species-specific cell susceptibility to SARS-CoV-2, virus dose-dependent cytopathic effects, and infection kinetics between primary porcine and human respiratory epithelial cells expressing angiotensin-converting enzyme 2 (ACE2) receptor over the course of infection. In addition, we identified a potential mechanism for self-limiting SARS-CoV-2 infection in porcine respiratory epithelial cells.

## Results

### Distribution of ACE2 receptor on porcine and human tracheal epithelial cells

**Fig. 1:**
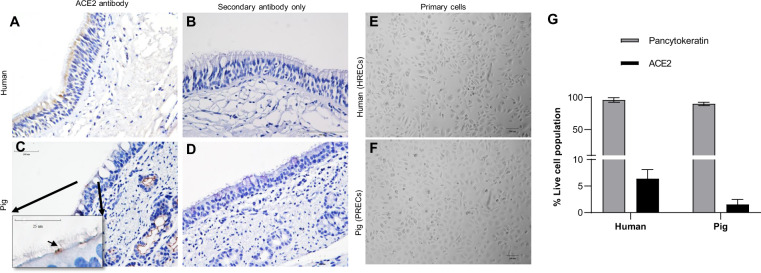
Distribution of ACE2 receptor on PRECs and HRECs, and corresponding trachea tissue sections. **A**–**D** Formalin-fixed paraffin-embedded cross-sections of human and pig tracheal sections stained for ACE2 with ImmPRESS VR anti-mouse IgG horseradish peroxidase (HRP) polymer detection kit (MP-6402-15). Brown spots represent the presence of ACE2 receptor that interacted with specific antibody is considered a positive expression, and the nucleus counterstained with hematoxylin is blue. Expression of ACE2 in human (**A**) and pig (**C**) trachea revealed by immunostaining with mouse anti-ACE2 monoclonal antibody (4 μg/mL; sc-390851). Note the focal expression of ACE2 on the epithelial cells of pig trachea (**C**; arrow in inset image). **B**, **D** Corresponding negative control tissues sections stained with secondary anti-mouse HRP antibody only. Scale bar-100 μm (inset 25 μm). Pig (*n* = 3); human (*n* = 1). Microscopic morphology of primary human (**E**; HRECs) and pig (**F**; PRECs) respiratory epithelial cells. **G** Determination of pan-cytokeratin and ACE2 in HRECs and PRECs using flow cytometry. Cells stained with LIVE/DEAD^®^ Fixable Near-IR Dead Cell Stain Kit, and respective mouse monoclonal antibodies (epithelial cell marker pan-cytokeratin; 0.5 μg/mL; MCA1907T); (ACE2; 4 μg/mL; sc-390851,) incubated for 30 min followed by incubation with Alexa Fluor^®^ 647 Goat anti-mouse (15 μg/mL) for another 30 min. Data was collected using an Attune NxT flow cytometer. A representative of 10,000 events were acquired and analyzed for each sample. Cells were gated for singlet population using forward (FSA) and side-scatter (SSA) properties, and the mean of percent live cell population was used to quantify the levels of pan-cytokeratin and ACE2 (*n* = 4). The bar graph represents the mean (SD).

Immunohistochemistry (IHC) analysis of the ACE2 expression on tissue slides corresponded to normal adult human trachea tissue sections of commercially acquired slides indicated that ACE2 expression was predominantly present on epithelial cells, particularly towards the tracheal epithelial lining (Fig. 1A). However, ACE2 expression was not observed in the subepithelial region of the trachea. In the pig tracheal section collected for this study, ACE2 was intermittently expressed on the epithelial cells, while its expression was predominantly observed on the epithelial lining of submucosal glands (Fig. 1C). Even the subcultured primary cells isolated from human and pig tracheal epithelium showed evident expression of pan-cytokeratin and ACE2 (Fig. 1G). However, a relative quantification of ACE2 expression by flow cytometric analysis, revealed that ACE2 expression level on PRECs was comparatively lower than that in HRECs (Fig. 1G).

### SARS-CoV-2 binding on human and pig tracheal epithelium and its infection in HRECs and PRECs

**Fig. 2:**
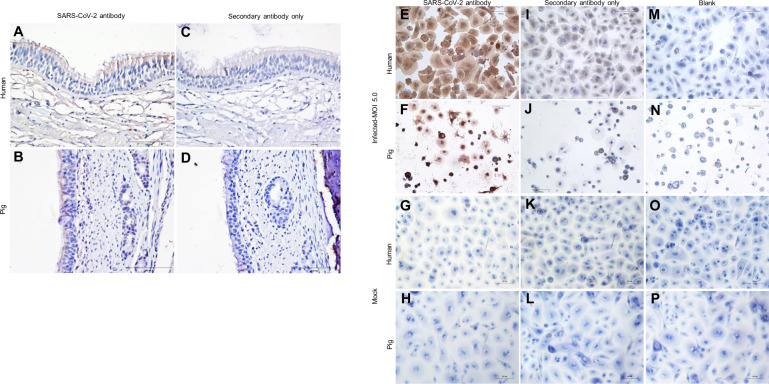
SARS-CoV-2 binding on human and pig tracheal epithelium and infection in HRECs and PRECs. The tissues and cells presented in this panel were stained with ImmPRESS VR anti-rabbit IgG horseradish peroxidase (HRP) polymer detection kit (MP-6401-15) with a rabbit monoclonal antibody specific for anti-SARS Coronavirus/SARS-Related Coronavirus 2 nucleocapsid (N) protein (0.75 μg/mL) (the following reagent was obtained through BEI Resources, NR-53791). Dark brown spots represent a positive expression of viral N protein, and pale brown represents background staining, while the nucleus counterstained with hematoxylin is blue. **A**–**D** Representative images showing SARS-CoV-2 binding in formalin-fixed paraffin-embedded cross-sections of **A** human and **B** pig trachea after overnight incubation with 250 μL heat-inactivated SARS-Related Coronavirus 2, Isolate USA-WA1/2020 (the following reagent was obtained through BEI Resources; NR-52286). **C** Human (*n* = 1) and **D** pig (*n* = 3) tracheal epithelial sections were stained with secondary anti-rabbit HRP antibody only. **E**–**P** Detection of SARS-CoV-2 infection in HRECs and PRECs inoculated with SARS-CoV-2, Isolate USA-WA1/2020 at 120 hpi. Paraformaldehyde (4%) fixed cells were stained for SARS-CoV-2 N protein. **E**, **I**, and **M** HRECs and **F**, **J**, and **N** PRECS were inoculated with a viral dose of MOI 5.0 or culture medium, as negative control (**G**, **K**, and **O** for HRECs; **H**, **L**, and **P** for PRECs); (*n* = 6). Scale bar—100 μm.

Deparaffinized and heat-retrieved (citrate buffer) tissue sections of human and pig trachea incubated overnight with heat-inactivated SARS-CoV-2 (USA-WA1/2020) showed evidence of virus attachment. In the human trachea, the virus appeared to be bound on both the epithelial and subepithelial regions (Fig. 2A), while the binding was more confined to the epithelial lining in the pig trachea (Fig. 2B). In both human (Fig. 2C) and pig (Fig. 2D) trachea, the secondary antibody controls showed minimal background staining. Meanwhile, HRECs (Fig. 2E) and PRECs (Fig. 2F) inoculated with SARS-CoV-2 virus stock [dose multiplicity of infection (MOI) 5.0] propagated in Vero-E6 cells showed positive brown staining for rabbit anti-SARS-CoV-2 N protein monoclonal antibody by IHC staining. The corresponding mock-inoculated controls (Fig. 2G, H) remained negative throughout the infection. Cells stained with secondary antibody control showed minimum background staining (Fig. 2I–L), and blank controls without primary and secondary antibodies (Fig. 2M–P) were negative.

### SARS-CoV-2 infection and associated cell death in HRECs and PRECs

**Fig. 3:**
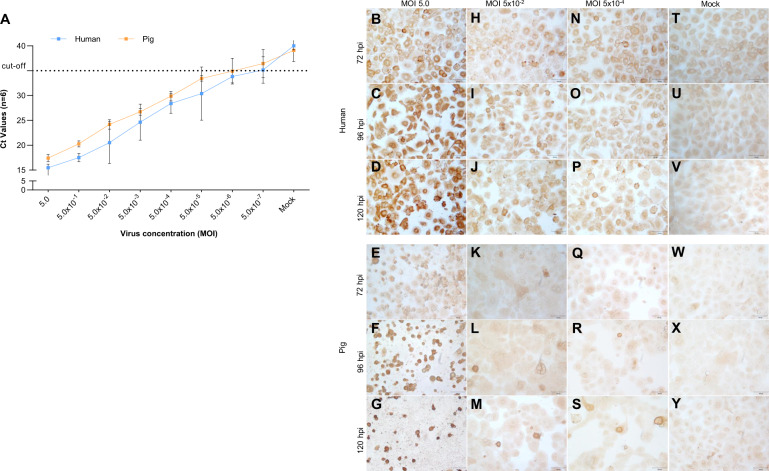
Dose-dependent infectious response of SARS-CoV-2 in HRECs and PRECs. **A** Detection of SARS-CoV-2 viral nucleocapsid (N) gene using EZ™-SARS-CoV-2 Real-Time RT-PCR developed by Tetracore. A volume of 7 μL Trizol extracted viral RNA sample was used in each reaction, and all RT-qPCR reactions were set up by including negative, positive, and no-template controls (NTC). Data from 6 technical replicates at each infection dose. Blue—HRECs (Human), Orange—PRECs (Pigs) (*n* = 6). **A** Line graphs (Mean; SD) plotted using RT-qPCR Ct values obtained from the supernatants of HRECs and PRECs inoculated with various doses of SARS-CoV-2 (Isolate USA-WA1/2020) for 120 h. **B**–**Y** Cells fixed in 4% paraformaldehyde were stained for SARS-CoV-2 viral N protein with ImmPRESS VR anti-rabbit IgG horseradish peroxidase (HRP) polymer detection kit (MP-6401-15; Vector Laboratories) and a recombinant anti-SARS-CoV-2 N protein rabbit monoclonal antibody (0.75 μg/mL) [The following reagent was obtained through BEI Resources, NIAID, NIH: Monoclonal Anti-SARS Coronavirus/SARS-Related Coronavirus 2 Nucleocapsid Protein (produced in vitro), NR-53791; SinoBio Cat: 40143-R001]. Dark brown represents a positive expression of the antibody, and pale brown represents background staining. HRECs inoculated with SARS-CoV-2 at three different infectious doses, i.e., MOI 5.0 (**B**–**D**), 5.0 × 10^−2^ (**H**–**J**), 5.0 × 10^−4^ (**N**–**P**) and mock (**T**–**V**), and three different time points [(**B**, **H**, **N**, **T**) 72 hpi; (**C**, **I**, **O**, **U**) 96 hpi; (**D**, **J**, **P**, **V**) 120 hpi]. Similarly, PRECs inoculated with SARS-CoV-2 at **E**–**G** MOI 5.0, **K**–**M** 5.0 × 10^−2^, **Q**–**S** 5.0 × 10^−4^, and **W**–**Y** mock-inoculated with culture medium, as well as three different time points [**E**, **K**, **Q**, **W** 72 hpi; **F**, **L**, **R**, **X** 96 hpi; **G**, **M**, **S**, **Y** 120 hpi]. Scale bar—100 μm.

Both PRECs and HRECs cultures were SARS-CoV-2-inoculated with eight different viral doses (MOI 5.0, 5.0 × 10^−1^, 5.0 × 10^−2^, 5.0 × 10^−3^, 5.0 × 10^−4^, 5.0 × 10^−5^, 5.0 × 10^−6^, and 5.0 × 10^−7^) or mock-inoculated with inoculation medium, and incubated for 120 h post-inoculation (hpi). The average Ct values at the highest dose used (MOI 5.0) in HRECs and PRECs after 120 hpi were 15.5 and 17.5, respectively (Fig. 3A). Approximately an average of three Ct increase (less virus) was detected for every 10-fold virus dilution until MOI 5.0 × 10^−5^, and the dilutions below MOI 5.0 × 10^−6^ were near or above the threshold cut-off of 35 (Fig. 3A).

Following infection, significant cytopathic changes such as rounding of cells, cell detachment, and vacuolation were observed by 72 hpi in both PRECs and HRECs. Overall, the cytopathic effects (CPE) increased with time, and SARS-CoV-2 infection in PRECs and HRECs is dose-dependent. The CPE was more evident at a high viral dose (MOI 5.0) by 72 hpi (Fig. 3B, E). Compared to HRECs, the cell death/cell detachment remarkably increased in PRECs at a viral dose of MOI 5.0 by 96 hpi (Fig. 3C, F) and continued through 120 hpi (Fig. 3D, G). However, no significant differences in HRECs and PRECs death/cell detachment were noticed between viral doses MOI 5.0 × 10^−2^ (Fig. 3H–M), 5.0 × 10^−4^ (Fig. 3N–S), and mock (Fig. 3T–Y). The cell detachment could be attributed to virus-induced cell death. Therefore, the potential role of apoptosis (nuclear fragmentation assay) and cytotoxicity (dual apoptosis/toxicity assay) was examined in HRECs, and PRECS inoculated with SARS-CoV-2 at MOI 5.0 or mock-inoculated with culture medium, and incubated for 120 hpi.

**Fig. 4:**
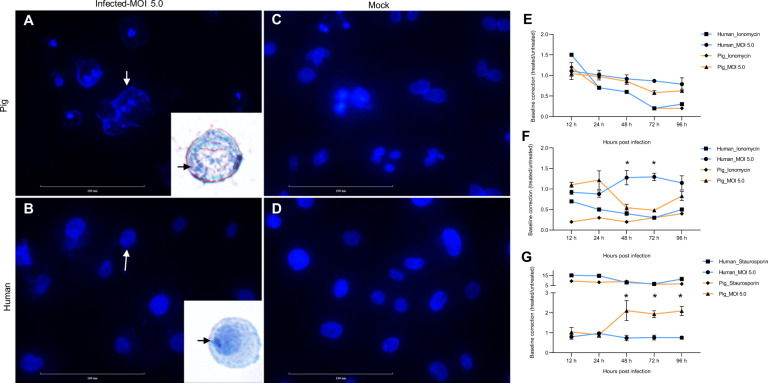
SARS-CoV-2 associated cell death in HRECs and PRECs. **A**–**D** Representative images of 4% paraformaldehyde-fixed cells stained with NucBlue fixed cell ReadyProbes reagent (DAPI) showing cell nuclear morphology in **A** PRECs and **B** HRECs treated with SARS-CoV-2 (Isolate USA-WA1/2020) at MOI 5.0 for 9 h. Note the differences (arrows) in nuclear condensation and fragmentation (a hallmark of apoptosis) between human and porcine cells; inset images were stained with hematoxylin. **C** PRECs and **D** HRECs mock-inoculated cells; Scale bar-100 μm; (*n* = 6). **E**–**G** Line graphs (Mean; SD) were generated using ApoTox-Glo triplex assay data. The relative fluorescence/luminescence units obtained from SARS-CoV-2 inoculated (treated) samples were normalized against their respective mock-inoculated control (culture medium) at each time point. Both HRECs and PRECs were inoculated with SARS-CoV-2 at MOI 5.0, 25 µM of ionomycin, and 0.625 µM of staurosporine for 12, 24, 48, 72, and 96 hpi. **E** Viability assay measuring live-cell protease activity, **F** Cytotoxicity assay measuring dead-cell protease activity, **G** Apotox assay measuring cleaved Caspase-3/7; (*n* = 3). *p-value < 0.05.

Cells stained with DAPI or hematoxylin revealed cell nuclear fragmentation only in SARS-CoV-2 infected PRECs (Fig. 4A), while the nucleus remained intact in HRECs infected cells (Fig. 4B) and mock-inoculated control cells from both species (Fig. 4C, D). Nuclear fragmentation is the hallmark feature in apoptotic cells. This finding was further confirmed using the ApoTox assay that measures cell viability, cytotoxicity, and apoptotic activity in the cells. The data of relative fluorescence/luminescence units obtained from HRECs and PRECs inoculated with SARS-CoV-2 were normalized against their respective mock-inoculated cell controls at each time point through the infection period. The positive controls ionomycin (Fig. 4E, F) and staurosporine (Fig. 4G) performed as expected. The cell viability gradually decreased with time (12-96 hpi) in HRECs and PRECs inoculated with the virus (Fig. 4E). However, the SARS-CoV-2-mediated decrease in cell viability observed in HRECs and PRECs following virus infection resulted in two different outcomes: HRECs infection was driven by a cytotoxicity-mediated mechanism (Fig. 4F), while PRECs infection was mediated by apoptosis (Fig. 4G).

### SARS-CoV-2 replication kinetics in HRECs and PRECs

**Fig. 5:**
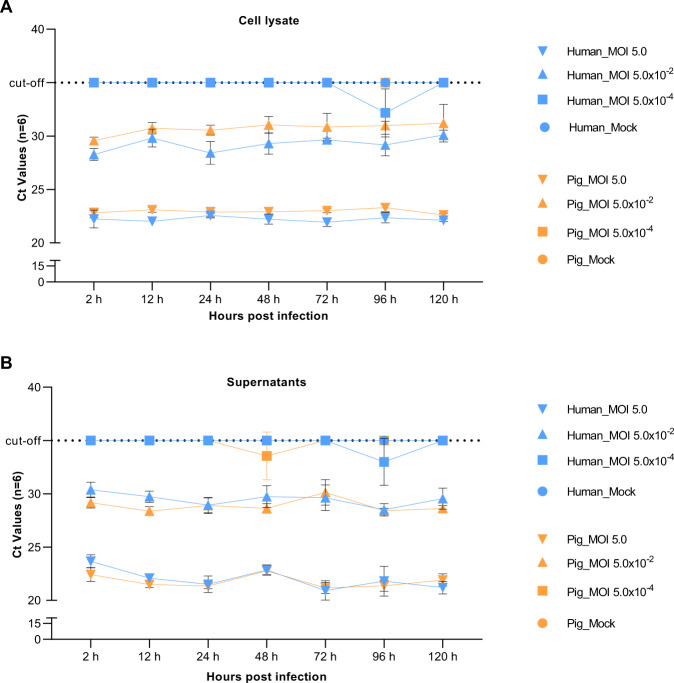
Analysis of SARS-CoV-2 replication in cell lysates and supernatants HRECs and PRECs. Detection of SARS-CoV-2 viral nucleocapsid (N) gene using EZ™-SARS-CoV-2 Real-Time RT-PCR developed by Tetracore. A volume of 7 μL Trizol extracted viral RNA sample was used in each reaction, and all RT-qPCR reactions were set up by including negative, positive, and no-template controls (NTC). Data from 6 technical replicates at each dose. Blue—HRECs (human), Orange—PRECs (pigs) (*n* = 6). **A** Line graphs showing viral growth over time in cell lysates and **B** the supernatants of HRECs and PRECs inoculated with SARS-CoV-2 at MOI 5.0, 5.0 × 10^−2^, 5.0 × 10^−4^, or mock-inoculated with culture medium.

To evaluate the SARS-CoV-2 viral replication kinetics in PRECs and HRECs culture supernatants from six replicates were collected at different time points (2, 12, 24, 48, 72, 96, 120 hpi) for each inoculated viral dose and evaluated using a commercial SARS-CoV-2 *N* gene-based RT-qPCR assay. Based on the dose–response data previously presented herein, three different virus doses (MOI 5.0, 5.0 × 10^−2^, 5.0 × 10^−4^) were selected to further evaluate the virus replication (viral load) kinetics in HRECs and PRECs by IHC and RT-qPCR. No significant differences in Ct values were observed between HREC and PREC virus-infected lysates (Fig. 5A) and supernatants (Fig. 5B) collected at 2, 12, 24, 48, 72, 96, 120 hpi. An average of six Ct increase was noticed between MOI 5.0 and 5.0 × 10^−2^, while at MOI 5.0 × 10^−4^, the Ct values were near or above the cut-off Ct value (35 cycles). In addition, IHC for SARS-CoV-2 N protein revealed the production of viral proteins in HRECs (Fig. 3B–D, H–J, and N–P) and PRECs (Fig. 3E–G, K–M, and Q–S) infected with SARS-CoV-2, being more evident at MOI 5.0 in both HRECs (Fig. 3B–D) and PRECs (Fig. 3E–G). In comparison, mock-inoculated HRECs (Fig. 3T–V) and PRECs (Fig. 3W–Y) stained negative.

### Supernatants collected from PRECs previously inoculated with a SARS-CoV-2 were not infectious

**Fig. 6:**
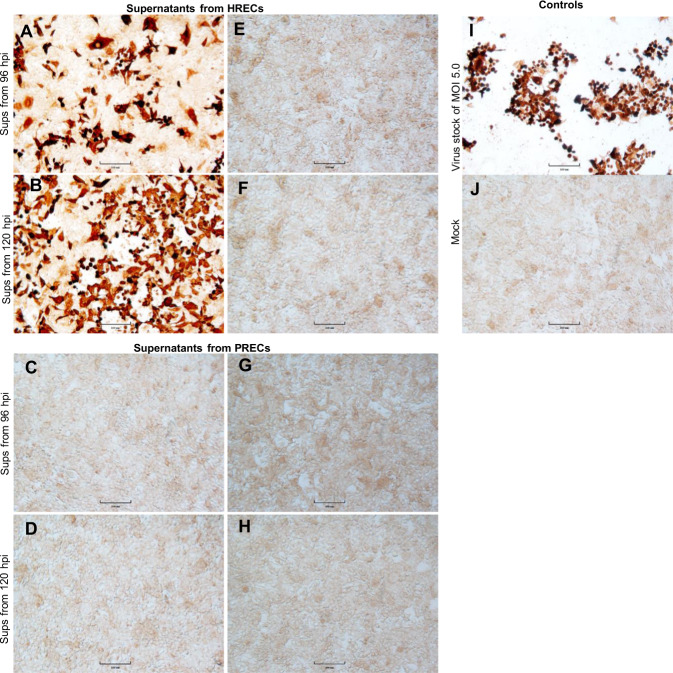
Supernatants collected from PRECs previously inoculated with a SARS-CoV-2 were not infectious. Immunocytochemistry images of 4% paraformaldehyde-fixed Vero-E6 cells showing the expression of SARS-CoV-2 N protein. Cells stained with ImmPRESS VR anti-rabbit IgG horseradish peroxidase (HRP) polymer detection kit (MP-6401-15) and a recombinant anti-SARS-CoV-2 N protein rabbit monoclonal antibody (0.75 μg/mL) (the following reagent was obtained through BEI Resources; NR-53791). Dark brown spots represent a positive expression, and pale brown spots represent background staining. Scale bar—100 μm. **A**–**J** Vero-E6 cells inoculated with supernatants from **A**, **B** HRECs and **C**, **D** PRECs previously infected with SARS-CoV-2 (MOI 5.0) and their corresponding supernatants from **E**, **F** HRECs and **G**, **H** PRECs mock-inoculated with culture medium (negative controls). The **A**, **E**, **C**, and **G** panels correspond to supernatants collected at 96 hpi, while **B**, **F**, **D**, and **H** panels correspond to supernatant collected at 120 hpi. **I** Vero-E6 cells inoculated with SARS-CoV at MOI 5.0, from the original SARS-CoV-2 virus stock, used as IHC positive control, and **J** corresponding mock-inoculated control.

The potential infectivity of supernatants collected from HRECs and PRECs cultures previously infected (MOI 5.0) with SARS-CoV-2 was assessed on Vero-E6 cells. The virus replicated efficiently in Vero-E6 cells when cell culture supernatants collected at 96 hpi and 120 hpi from HRECs were used as inoculum (Fig. 6A, B). In contrast, no virus replication was observed in Vero-E6 cells when the inoculum used was originated from PRECs previously infected with MOI 5.0 SARS-CoV-2 (Fig. 6C, D). Mock-inoculated supernatants collected from respective time points and cell types were used as controls (Fig. 6E–H). For staining controls, Vero-E6 treated with original stock (MOI 3.3) was used as a positive control (Fig. 6I), and mock inoculation media was used as a negative control (Fig. 6J).

## Discussion

The ongoing global pandemic of SARS-CoV-2 has resulted in different clinical outcomes in two closely related mammalian species, humans and pigs. While in humans, the COVID-19 pandemic has resulted in >4.5 million deaths across the world (>223 million confirmed cases; as of 09/10/2021) [26], pigs, in contrast, and according to previous reports, seem to be either not susceptible to SARS-CoV-2 infection [14, 22, 23] or where the infection is mild and self-limited [25].

The successful reproduction of infection and clinical disease in vivo under experimental settings can be difficult in pigs, even with swine-restrictive viruses. Constraints include resource-intensive, susceptibility-related factors, inoculum dose and route of exposure, high variability, lack of sensitivity, interference with gut microbiome or secondary infections, and difficulty recording precise cell–viral interactions on a daily/hourly basis. In contrast, in vitro, culture models based on cell lines are relatively easy to maintain, but often they are not the natural cell target of the virus, nor do they represent sufficient complexity (cell lineage, functionality) to mimic the natural infection process in vivo [27]. For this reason, in vitro experimental data cannot often be extrapolated into clinical trials entirely, e.g., complicated cellular signals between cells and their matrix cannot be reproduced [28]. This would justify using primary respiratory epithelial cell cultures to understand the immunopathogenesis of SARS-CoV-2.

Tracheobronchial-derived primary epithelial cells have been widely used to study early immune responses towards viral infections [29–32]. Thus, the first objective of this study was to confirm whether porcine respiratory epithelial cells were susceptible to infection by SARS-CoV-2 and comparing it with human respiratory epithelial cells.

Using ACE2 as entry receptor and proteases as entry activators [33], SARS-CoV-2 spike protein mediates virus entry into the respiratory epithelial cells of a susceptible host, where the virus primarily replicates [34, 35]. Previous studies reported the detection of viral antigens in the human trachea, bronchi, bronchiole, and pneumocytes [36] tracheal degeneration and necrosis in affected cats [14], alveolar damage and necrosis in minks [37], and detection of viral RNA in the bronchi of white tailed-deer [20].

First, the present study demonstrated the expression of ACE2 receptor and effective SARS-CoV-2 binding on the epithelial lining of both human and pig tracheal tissue sections, which contradicts a previous study that hypothesized that the lack of virus susceptibility or virus replication could be attributed to the absence of ACE2 receptors on the porcine respiratory tract epithelium [38]. This finding is supported by a recent study showing that ACE2 is expressed, at different levels, in a wide range of porcine tissues, including the lungs [39].

In our study, a human ACE2 antibody was used for IHC analysis on both human and pig trachea tissue sections and primary respiratory cells. The expression levels of ACE2 were significantly low on pig tracheal epithelium tissue sections compared to their human counterparts (Fig. 1A, C). Further, flow cytometric analysis of isolated PRECs and HRECs quantified the amount of both pan-cytokeratin and ACE2 expressed on these cell types, confirming that human cells expressed more ACE2 than pig cells. Despite the paucity in ACE2 expression on pig trachea, heat-inactivated SARS-CoV-2 bound uniformly across the pig and human trachea tracheal epithelium (Fig. 2A, B).

The protein sequence homology studies between human ACE2 (NP_001358344) and pig ACE2 (NP_001116542) performed in this study and others [40] suggest that these closely related mammalian species share 81% identical amino acid residues. Further, a pair-wise alignment of porcine ACE2 protein with human ACE2 protein at the region targeted by the anti-ACE2 antibody (amino acids 631-805; sc-390851, Santa Cruz Biotechnology) used in this study shows a sequence similarity of 76% (Supplementary Fig. 1), suggesting the potential cross-reactivity and usefulness of anti-ACE2 antibody towards detection of porcine ACE2 receptor in tissues and cultures.

After confirming the expression of ACE2 receptors in human and pig tracheobronchial-derived tissue sections and cells, we performed a comparative in vitro infection study to investigate possible factors related to possible differences in the susceptibility of primary porcine and human tracheobronchial epithelial cells to SARS-CoV-2 infection. Firstly, the optimal viral dose in PRECs and HRECs cultures was established for subsequent infection studies. As to the question of whether SARS-CoV-2 can infect and replicate in PRECs, SARS-CoV-2 replicated in both PRECs and HRECs in a dose-dependent manner, as evidenced by RT-qPCR and IHC assays (Fig. 3). Virus replication was monitored in PRECs and HRECs cultures inoculated with three different infectious doses (MOI 5.0, 5.0 × 10^−2^, and 5.0 × 10^−4^) over 120 hpi period. SARS-CoV-2 N protein was gradually accumulated in both PRECs and HRECs cultures as infection progressed, particularly at the higher infectious dose (MOI 5.0) used in this study. The CPE was particularly evident in PRECs compared to HRECs or the corresponding mock-inoculated controls. This CPE was dose- and time-dependent, dramatically enhanced in PRECs at MOI 5.0 infectious dose and 96 hpi (Fig. 3E–G). This strongly indicates that virus dose is a potential factor in the outcome of SARS-CoV-2 infection in these primary cells, as it was previously hypothesized [41]. Previous in vivo studies in pigs demonstrated that only using a high infectious dose (2 mL of approximately 10^6^ TCID50/mL intranasally and intratracheally) triggered the production of anti-SARS-CoV-2 neutralizing antibodies even in the absence of clinical signs [24, 25].

Subsequently, we investigated the overall mechanism behind the CPE and massive cell death particularly observed in PRECs cultures inoculated with the higher infectious dose (MOI 5.0) evaluated in this study. In general, CPE and cell death could be either cell-associated (i.e., cells died because of their inability to reproduce) or virus-induced (i.e., lysis and dissolution caused by virus infection). This can be elucidated on the basis of general morphological, biochemical, and functional features [42].

Specifically, morphological analysis of SARS-CoV-2 infected PRECs cultures revealed all the hallmark morphological signs of apoptosis, a controlled form of cell death [42], including cell shrinkage and detachment, plasma membrane blebbing, the formation of apoptotic bodies, chromatin condensation (pyknosis), and nuclear fragmentation (karyorrhexis) leading to cell death (Fig. 5C). In contrast, in HREC cultures, most cells were attached to the plate and appeared viable with no notable differences in the morphology of the nucleus between SARS-CoV-2- and mock-inoculated cultures (Fig. 5A, B).

On the other hand, the genetic and biochemical cell-death analysis includes activation of cysteine aspartate-specific proteinases (caspases) and releasing mitochondrial factors as crucial features of the apoptotic process [42]. Using the biochemical ApoTox-Glo triplex assay, we further demonstrated an early and enhanced apoptotic mechanism mediated through caspase 3/7 activation in response to SARS-CoV-2 infection in PRECs compared to HRECs. The decrease in cell viability was particularly high in SARS-CoV-2 infected PRECs after 48 hpi (Fig. 4G). Contrary, the expression levels of caspase 3/7 in HRECs infected cultures remained stable throughout the infection period. Additional SARS-CoV-2 infection studies on human bronchial epithelial cells (BEAS-2B) also reported no induction of apoptosis [43]. Interestingly, SARS-CoV-2 induced enhanced cytotoxicity in HRECs compared to PRECs cultures after 48 hpi (Fig. 4F). In addition, the supernatants collected from HRECs infected with SARS-CoV-2 contained infectious virions that were able to infect Vero-E6 cells, while the corresponding supernatants from PRECs undergo apoptosis lacked viable virus and were, therefore, non-infectious. In 2012, Nelli and others reported comparable findings in primary human and porcine respiratory epithelial cells infected with highly pathogenic H5N1 influenza A virus (IAV) [31, 44].

The results of the present study demonstrated that an early onset of apoptosis via caspase 3/7 activation is a crucial event to limit SARS-CoV-2 propagation in PRECs. Thus, further research on modulation of apoptosis and the effect of caspase inhibitors is needed. The early apoptotic cell death observed in PRECs may favor the host cell, while the delayed cell death observed in HRECs may favor the virus. Previous experimental studies in vivo observed complete virus (RNA) clearance one week after virus inoculation in pigs [45]. This, together with additional studies reporting absence of clinical signs and effective virus transmission between animals [14, 22–25], led to conclude that pigs are more resistant to SARS-CoV-2 infection than humans and other animal species such as cats, mink and deer.

Taken together, our findings shed light on the possible molecular mechanism of resistance of pigs to SARS-CoV-2 infection and/or virus propagation, and it may hold therapeutic value for the treatment of COVID-19.

## Material and methods

All infection experiments involving SARS-CoV-2 were performed in the BSL-3 laboratory facilities at Iowa State University (ISU) under pre-established/approved protocols.

### Tissue collection and isolation of primary porcine respiratory epithelial cells

Tissues from the tracheal region of the respiratory tract, i.e., from below the larynx to the bronchial bifurcation (approx. 6–8 inches), were aseptically collected from 7-day-old healthy cesarean-derived, colostrum-deprived (CD/CD) pigs (Yorkshire x Large White crossbred, Struve Labs International, Inc., Manning, IA, USA) immediately after necropsy. The experimental protocol for porcine sample collection was approved by the Institutional Animal Care and Use Committee (IACUC log# 12-17-8658-S; approval date: January 3, 2018) at ISU. Briefly, piglet tracheal sections were collected in Dulbecco’s Minimum Essential / Ham’s F-12 medium with GlutaMAX (DMEM/F-12) (Thermo Fisher Scientific, Waltham, MA, USA), supplemented with 100 IU/mL of penicillin/100 µg/mL of streptomycin (Pen-Strep) (Thermo Fisher Scientific), and 1.25 µg/mL of amphotericin B (AmpB) (Thermo Fisher Scientific) for isolation of PRECs as previously described [32]. Tracheal samples were washed and incubated in phosphate-buffered saline (PBS) pH 7.4 supplemented with Pen-Strep to remove any blood clots. Then, samples were incubated at 4 °C for 48 h in digestion medium [calcium and magnesium-free Minimum Essential Medium (MEM; in-house), supplemented with 1.4 mg/mL pronase (Millipore-Sigma, Burlington, MA, USA), 0.1 mg/mL DNase (Millipore-Sigma), 100 µg/mL primocin (Invivogen, San Diego, CA, USA). Tissue digestion was neutralized using 10% heat-inactivated EqualFetal fetal bovine serum (FBS; Atlas Biologicals, Fort Collins, CO, USA). The tissue digest containing cells was passed through a 40 µm cell strainer, washed, pelleted, and resuspended in DMEM/F12 medium. Collected cells were either seeded directly using respective growth medium or frozen in LHC^®^ basal medium (Thermo Fisher Scientific) containing 30% FBS and 10% dimethyl sulfoxide (DMSO) (Millipore-Sigma). A portion of the tissue sections was fixed in 10% buffered neutral formalin for IHC analysis.

### Culture of primary porcine and human respiratory epithelial cells

Both isolated primary PRECs and commercially acquired HRECs (ATCC, PCS-300-010, Lot-70002486) were subcultured on cell/tissue culture flasks or plates (Greiner Bio-One North America Inc, Monroe, NC, USA), pre-coated with PureCol^®^ Type I collagen (40 µg/mL/4 mm^2^; Advanced BioMatrix, Inc., San Diego, CA, USA), at a density of ~20,000 cells/ cm^2^. Both PRECs and HRECs were propagated in ATCC airway epithelial cell basal medium (ATCC^®^ PCS-300-030™) supplemented with 500 mg/mL HSA, 0.6 mM linoleic acid, 0.6 mg/mL lecithin, 6 mM L-Glutamine, 0.4% Extract P, 1.0 mM epinephrine, 5 mg/mL transferrin, 10 nM 3,3′,5-Triiodo-L-thyronine (T3), 5 mg/mL hydrocortisone, 5 ng/mL rh epidermal growth factor (EGF), 5 mg/mL rh insulin, Pen-Strep and Amp-B (growth media). Cells were dissociated with 0.5X TrypLE^TM^ express enzyme (Thermo Fisher Scientific) and neutralized using 50% heat-inactivated FBS (EquaFetal™, Atlas Biologicals), mixed in LHC basal medium (Thermo Fisher Scientific). Specifically, primary cells used in this study corresponded to passage 16 for PRECs and 9 for HRECs.

### SARS-CoV-2 culture and propagation in vitro

Vero-E6 cells (ATCC, CRL-1586) were used to propagate SARS-CoV-2 (the following reagent was deposited by the Centers for Disease Control and Prevention and obtained through BEI Resources, NIAID, NIH: SARS-Related Coronavirus 2, Isolate USA-WA1/2020, NR-52281) according to CDC protocol [46]. In brief, cells were sub-cultured in DMEM (Corning, Tewksbury, MA, USA) supplemented with 10% FBS. After culturing the trypsinized cells for 24 h, the cells were treated with SARS-CoV-2 virus at 0.05 plaque-forming units (PFU)/cell. The inoculated cultures were then incubated at 37 °C in humidified 5% CO_2_ incubator and observed for viral replication and CPE daily. Viral supernatants were collected from culture flasks showing CPE greater than 90%, and after removing the cell debris, the virus titer in the supernatants was determined by plaque assay in Vero-E6 cells. After propagating for 3 passages, approximately 10^7^ PFU/mL of virus titer was achieved, which were aliquoted and frozen at −80 °C for subsequent virus infectious studies on HRECs and PRECs.

### SARS-CoV-2 titration in Vero-E6, HRECs, and PRECs

For virus titration assays, 20,000 (Vero-E6/ HRECs/ PRECs) cells were seeded per well in a 96-well plate. In both PRECs and HRECs, before the day of infection, the cells were washed once with LHC media and pre-incubated with an infection medium containing ATCC airway epithelial cell basal medium, 2% Ultroser-G (Sartorius Stedim Biotech GmbH, Goettingen, Germany), 1×4-(2-hydroxyethyl)−1-piperazineethanesulfonic acid (HEPES) (Thermo Fisher Scientific), 1X MEM non-essential amino acids (Thermo Fisher Scientific), 1X Glutamax (Thermo Fisher Scientific), Pen-Strep and AmpB for 24 h. The cells were washed once with LHC medium on the day of infection and replaced with infection media containing different doses of SARS-CoV-2 (MOI 5.0, 5.0 × 10^−1^, 5.0 × 10^−2^, 5.0 × 10^−3^, 5.0 × 10^−4^, 5.0 × 10^−5^, 5.0 × 10^−6^, 5.0 × 10^−7^) and mock inoculum. A volume of 100 µL of viral stock/supernatants with along with 100 µL of inoculation media was used as inoculum. After 2 h incubation at 37 °C and 5% CO_2_, the virus and mock inoculum were removed, cells were washed once with LHC medium and replaced with fresh infection media and incubated for 2-, 12, 24, 48, 72, 96, or 120 hpi, respectively. Following infection, the cells on plates were either fixed in 4% paraformaldehyde for imaging or lysed in Trizol for RNA isolation, while the supernatants were directly collected into Trizol for viral RNA extraction.

### Immunohistochemistry staining in tissues, HRECs, and PRECs

Immunohistochemistry staining was used to confirm the expression of the viral host receptor for SARS-CoV-2, ACE2 (human ACE2 amino acids 631-805; sc-390851, Santa Cruz Biotechnology) in paraffin-embedded tracheal tissue sections, and primary HRECs and PRECs. Normal human trachea tissue slides were commercially bought (Novus, NBP2-77809 Novus Biologicals, LLC, Centennial, CO, USA), while pig trachea sections were collected for this study. Deparaffinized sections were heat retrieved (96° C/30 min) using citrate buffer (Millipore-Sigma) and washed in tris-buffered saline containing 0.1% Tween 20 (TBST) (Millipore-Sigma). In HRECs and PRECs, confluent cells on plates were fixed with 4% paraformaldehyde for 15 min and subsequently permeabilized with 0.1% Triton X-100 (Millipore-Sigma) for 10 min. The following primary antibodies were used in this study, mouse anti-ACE2 (4 μg/mL; E-11; sc-390851; Santa Cruz Biotechnology, Dallas, TX, USA); mouse anti-pan-cytokeratin (0.5 μg/mL; AE1/AE3; MCA1907T; Bio-Rad Laboratories, Hercules, CA, USA); and a recombinant anti-SARS-CoV-2 nucleocapsid (N) protein rabbit monoclonal antibody (0.75 μg/mL) [The following reagent was obtained through BEI Resources, NIAID, NIH: Monoclonal Anti-SARS Coronavirus/SARS-Related Coronavirus 2 Nucleocapsid Protein (produced in vitro), NR-53791; SinoBio Cat: 40143-R001].

Tissue sections or cells were stained using ImmPRESS VR anti-mouse/anti-rabbit IgG HRP polymer detection kit (MP-6402-15/ MP-6401-15; Vector Laboratories) was used as per the manufacturer’s instructions. In brief, sections/cells blocking with animal-free buffer (Vector Laboratories) for 30 min and incubated overnight with the corresponding mouse or primary rabbit antibody at 4 °C. The tissues were treated with 0.1% hydrogen peroxide for 1 h, while cells were treated for 5 min, followed by incubation with the respective secondary antibody for 60 min. Chromogenic detection in situ was performed using ImmPACT DAB EqV peroxidase substrate solution (Vector Laboratories) and hematoxylin, followed by mounting (tissue sections only) in Tissue-Tek Glas mounting medium (Sakura Finetek). Microscopic images were captured using Olympus^®^ CKX4 microscope (Olympus^®^ Corp., Center Valley, PA USA), Infinity 2 camera, and Infinity Analyze imaging software (Ver 6.5.5, Lumenera Corp, Ottawa, ON, Canada).

### Cellular characterization using flow cytometry

Confluent monolayers of HRECs and PRECs were trypsinized as described earlier, and dissociated cell suspension was incubated in PBS containing 100 μg/mL bovine deoxyribonuclease I (MilliporeSigma, St. Louis, MO, USA) and 5 mM magnesium chloride (MilliporeSigma) for 15 min at room temperature. After incubation, the cell suspension was passed through a 30 μm cell strainer (Miltenyi Biotec, Bergisch Gladbach, Germany), and cells were washed thoroughly by centrifuging at 200 x *g* for 5 min. Flow cytometric staining was performed using a cell concentration of approximately 200,000 cells per treatment in FACS buffer (PBS supplemented with 1% FBS and 0.09% sodium azide). After a 30 min incubation step on the ice, and washing twice with FACs buffer, the cells were stained with LIVE/DEAD™ Fixable Near-IR Dead Cell Stain Kit (Thermo Fisher Scientific) at a previously determined concentration of 1:200. For revealing ACE2 receptor expression, cells were stained with mouse anti-ACE2 (Santa Cruz Biotechnology) and fixed with BD Cytofix/Cytoperm™ solution (BD Biosciences, San Jose, CA, USA) for 20 min on ice. For assessing the pan-cytokeratin expression, fixed cells were permeabilized with Perm/Wash™ buffer (BD Biosciences) for 30 min on ice, washed and stained for mouse anti-pan-cytokeratin (Bio-Rad Laboratories). Then, after 30 min incubation on ice with a goat anti-mouse labeled to Alexa Fluor^®^ 647 (15 μg/mL, Jackson ImmunoResearch Laboratories, Inc., West Grove, PA, USA), the cells were washed twice and resuspended into 200 μL FACS buffer. Samples were analyzed on Attune NxT flow cytometer equipped with an autosampler (Thermo Fisher Scientific) as per manufacturer protocols, using appropriate threshold and gate settings. Each experiment samples tested in duplicate, including unstained, FMO, and isotype controls. Compensation controls were also performed, and the corresponding data were analyzed.

### Viral binding assay

Human and pig tracheal epithelial sections were incubated overnight at 37^o^C with 250 μL of heat-inactivated SARS-CoV- isolate USA-WA1/2020 (the following reagent was deposited by the Centers for Disease Control and Prevention and obtained through BEI Resources, NIAID, NIH: SARS-Related Coronavirus 2, Isolate USA-WA1/2020, Heat Inactivated, NR-52286) at 37 °C in a humidified chamber. After overnight incubation with the virus, the tissue sections were vigorously washed with TBST for 15 min, and the IHC staining was performed as described in the previous section.

### SARS-CoV-2 reverse transcriptase PCR (RT-qPCR) assay

Viral RNA extractions were performed using the E.Z.N.A.^®^ Viral RNA Kit (Omega Bio-tek, Inc., Norcross, GA, USA) and the vacuum manifold (QIAGEN, Germantown, MD, USA) method following the’manufacturer’s instructions. A SARS-CoV-2 viral *N* gene-based RT-qPCR developed and commercialized by Tetracore (Tetracore, Inc., Rockville, MD, USA) was used in this study as per recommended instruction manual and was modified for the use of the Rotor-Gene Q with the help of Tetracore. Each 25 μL RT-qPCR reaction contained: 16.75 μL EZ-SARS-CoV-2 Mastermix which included primers–probes for FAM-SARS-CoV-2, TAMRA-inhibition control in vitro transcript, Cy5-human RNase P; 0.5 μL inhibition control; 0.75 μL of enzyme; 7 μL of the extracted sample RNA. All RT-qPCR reactions included two positive controls, one supplied by the manufacturer and the other obtained through BEI Resources, NIAID, NIH (the following reagent was deposited by the Centers for Disease Control and Prevention and obtained through BEI Resources, NIAID, NIH; qPCR control RNA from heat-inactivated SARS-CoV-2 (isolate USA-WA1/2020, NR 52347), and a ““no template”” control (NTC). RT-qPCR reactions were run on a Rotor-Gene Q (QIAGEN) with cycling conditions, 48 °C for 15 min and 95 °C for 2 min holding; 40 cycles, 95 °C for 10 s denaturation, and 60 °C for 40 s amplification. The RT-qPCR results were analyzed using Rotor-Gene Q Pure Detection software (v 2.3.1). For this study, samples with a threshold cycle (Ct) above 35 were considered negative.

### Cell nuclear fragmentation assay

Paraformaldehyde (4%) fixed SARS-CoV-2 and mock-inoculated cells were permeabilized with 0.1% Triton X-100 for 10 min. Next, nuclear staining was performed using two drops/mL NucBlue fixed cell ReadyProbes reagent with ’4’, 6-diamidino-2 phenylindole (DAPI) (Thermo Fisher Scientific) in PBS and incubated for 5 min at room temperature. After washing thrice with PBS, microscopic images were captured using fluorescent microscopy (Olympus^®^ CKX4 microscope, Infinity 2 camera, and Infinity Analyze imaging software).

### Cell viability, cytotoxicity, and caspase 3/7 activity

The ApoTox-Glo™ Triplex Assay was used to assess the differences in cell death status (i.e., cell viability, cytotoxicity, and apoptosis) between HRECs and PRECs over the course (96 h) of SARS-CoV-2 infection. The live-cell protease activity restricted to intact viable cells was measured using a fluorogenic (400Ex/505Em; Viability), cell-permeant, peptide substrate (glycyl-phenylalanyl-aminofluorocoumarin; GF-AFC). A fluorogenic (485Ex/520Em; Cytotoxicity) cell-impermeant peptide substrate (bis-alanylalanyl-phenylalanyl-rhodamine 110; bis-AAF-R110) was used to measure dead-cell protease activity. A luminogenic caspase-3/7 substrate containing the tetrapeptide sequence DEVD is a reagent optimized for caspase activity, luciferase activity, and cell lysis was used to evaluate apoptosis. The cleavage of the inactive form of caspase to active caspases resulted in the luminescence signal produced by luciferase, which was proportional to the amount of caspase activity (apoptosis) present.

After seeding 15,000 cells per 96-well flat clear bottom black polystyrene surface-treated microplates (CellBind^®^; Corning), cells were inoculated with MOI 5.0 SARS-CoV-2 and mock controls and incubated for 12, 24, 48, 72, and 96 h, respectively. The ApoTox-Glo™ Triplex assay was performed as per manufacturer protocol. In brief, 20 μL of Viability/Cytotoxicity reagents containing both GF-AFC and bis-AAF-R110 substrates were added to all wells and mixed well and incubated for 30 min at 37 °C. Fluorescence was measured at two different wavelength sets. For caspase 3/7 activity, 100 μL of Caspase-Glo^®^ 3/7 reagent was added to all wells and mixed well and incubated for 30 min at room temperature, and the luminescence was immediately measured. Predetermined concentrations of staurosporine (0.625 μM; apoptosis control; Cayman Chemicals, Ann Arbor, MI, USA) and ionomycin (25 μM; viability and cytotoxicity control; Cayman Chemicals) as positive control were added to cell control wells 6 h before start recording the measurements at each time point. Measurements in all samples, i.e., infected, mock, and positive controls treated cells, were recorded in a POLARstar Omega microplate reader (BMG Labtech microplate reader BMG Labtech Inc., Cary, NC, USA).

### Data analysis

Statistical analyses and plots were performed using the data from flow cytometry, RT-qPCR, and ApoTox-Glo™ Triplex assays and analyzed using GraphPad Prism^®^ 9.0.2 software and Microsoft Excel. The statistical significance was determined using the two-way ANOVA multiple comparisons of Fisher’s least significant difference (LSD) test. For all analyses, a *p*-value <0.05 was considered statistically significant.

## Supplementary information

Supplementary Figure 1

Revised Manuscript - clean

Original Manuscript

## Data Availability

The original contributions presented in the study are included in the article/supplementary material, further inquiries can be directed to the corresponding author.
