# Supplementary figures and images for: Enhanced apoptosis as a possible mechanism to self-limit SARS-CoV-2 replication in porcine primary respiratory epithelial cells in contrast to human cells
Source: Cell Death Discov. 2021 Dec 10;7:383. doi: 10.1038/s41420-021-00781-w (PMC8661338; doi:10.1038/s41420-021-00781-w)

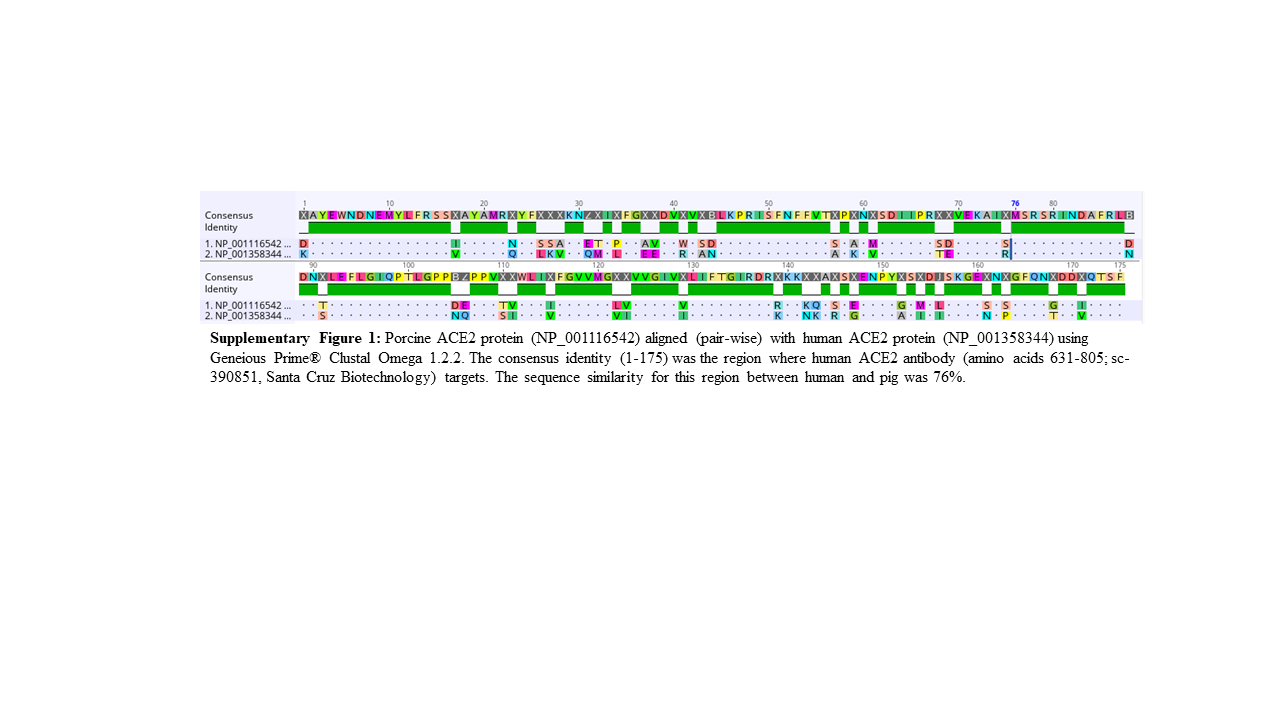

Supplement: Supplementary file 1 — Supplementary Figure 1 [file 41420_2021_781_MOESM1_ESM.tif]
